# Combined Nebulized and IV Tranexamic Acid for Hemoptysis Management: A Case Report and Brief Literature Review

**DOI:** 10.1002/ccr3.71443

**Published:** 2025-11-09

**Authors:** Erfan Ghadirzadeh, Faezeh Shokri, Shamim Khorshidian, Anita Ziari, Mobina Gheibi, Majidreza Adelani, Seyed Pooria Salehi Mashhad Sari, Fatemeh Varshoei, Hossein Mehravaran

**Affiliations:** ^1^ Student Research Committee, School of Medicine Mazandaran University of Medical Sciences Sari Iran; ^2^ Gastrointestinal Cancer Research Center, Non‐Communicable Diseases Institute Mazandaran University of Medical Sciences Sari Iran; ^3^ Department of Laboratory Sciences, Razi Hospital Mazandaran University of Medical Sciences Qaemshahr Iran; ^4^ Division of Pulmonary and Critical Care, Department of Internal Medicine, School of Medicine Mazandaran University of Medical Sciences Sari Iran; ^5^ Student Research Committee, School of Medicine Golestan University of Medical Sciences Gorgan Iran; ^6^ Department of Pediatrics, School of Medicine Mazandaran University of Medical Sciences Sari Iran

**Keywords:** case report, hemoptysis, literature review, nebulized, tranexamic acid

## Abstract

Hemoptysis management may necessitate invasive interventions, but combined nebulized and intravenous tranexamic acid (TXA) can play a promising noninvasive role in controlling hemoptysis. This case report describes two patients with non‐life‐threatening hemoptysis (a 71‐ and a 69‐year‐old man) successfully treated with combined nebulized and systemic IV TXA. A 71‐year‐old man with COPD exacerbation and non‐life‐threatening hemoptysis was successfully treated with TXA (both IV and nebulized) and standard care, which controlled the bleeding without adverse events or recurrence, and was discharged after 7 days. A 69‐year‐old male with bronchiectasis exacerbation experienced non‐life‐threatening hemoptysis. He was successfully treated with IV and nebulized TXA along with standard care, resolving the hemoptysis without recurrence and discharging the patient 8 days after admission. Existing evidence supports nebulized TXA's localized action, minimizing systemic absorption and thrombotic risks, though its efficacy on hemoptysis remains unproven. A brief review of 30 cases highlights nebulized TXA's high success rate with doses ranging from 250 to 2000 mg, though rare side effects like bronchospasm and serotonin‐like syndrome warrant caution. Playing a role as a potential alternative approach for antifibrinolytic administration in hemoptysis, especially for high‐thrombotic‐risk patients, and serving as a vital stabilization and bridging strategy before definitive interventional procedures, could be the main area for future studies.

## Graphical abstract

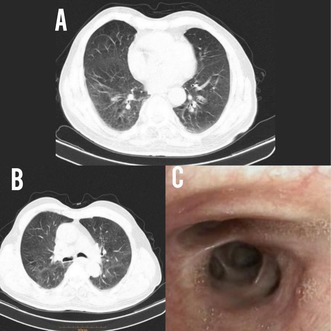

**Summary**

- Nebulized tranexamic acid may be used solely or as an adjunctive therapy in non‐massive hemoptysis to control the bleeding while reducing the adverse effects of systemic routes of administration; however, trials with good quality are needed to confirm such hypotheses.

## 1 Introduction

Hemoptysis ranges from mild to life‐threatening, with standard interventions like bronchoscopy, embolization, or surgical lobectomy, often requiring specialized resources [1]. Tranexamic acid (TXA), a systemic antifibrinolytic agent, can play a favorable role in controlling hemoptysis, considering its availability and cost‐effectiveness [2]. While systemic TXA is established for other bleeding conditions like postpartum hemorrhage [3], evidence for its efficacy in hemoptysis remains limited, though some trials suggest reduced bleeding duration [4].

While oral and intravenous (IV) routes have been the most commonly used methods for the administration of TXA, novel approaches have received clinical attention. Recent attention has shifted towards topical TXA administration (nebulized or endobronchial) as a potentially easier, faster, and more localized approach [5, 6], possibly reducing systemic side effects, especially beneficial in patients with contraindications like active thrombosis [7].

However, significant knowledge gaps persist. There is a paucity of robust literature evaluating nonsystemic TXA routes across diverse hemoptysis etiologies. Furthermore, the combined use of nebulized and systemic TXA lacks substantial investigation, creating an inconsistency between promising anecdotal reports and the lack of systematic evidence [7]. The theoretical rationale for this combination lies in simultaneously addressing hemoptysis with IV TXA for its foundational systemic effect while achieving high topical concentrations with nebulized TXA, potentially leading to faster hemostasis with a lower total systemic drug burden. Therefore, we describe the use of nebulized and systemic TXA simultaneously in the management of two airway disease patients with hemoptysis and review current reports of using topical TXA in hemoptysis treatment.

## 2 Case History/Examination

### 2.1 Case No. 1

A 71‐year‐old man presented to the emergency department (ED) of Sari, Imam Hospital, complaining of expectoration of sputum containing blood of one‐half cup volume and exacerbating dyspnea since one day before presentation, in addition to fever which started three days before admission. He had no complaints of nasal obstruction, epistaxis, pleuritic chest pain, hematemesis, bruises and trauma. The patient's past medical history (PMH) included chronic obstructive pulmonary disease (COPD), diabetes mellitus (DM) and hypertension (HTN), and a history of 20 pack‐year cigarette smoking.

### 2.2 Case No. 2

A 69‐year‐old male was admitted to Sari, Imam Hospital, with six episodes of non‐life‐threatening hemoptysis and exertional dyspnea one day before admission. His PMH included bronchiectasis, ischemic heart disease, DM and HTN. He had a smoking history exceeding 20 years but quit for the last 6 years.

## 3 Differential Diagnosis, Investigations, and Treatment

### 3.1 Case No. 1

**FIGURE 1:**
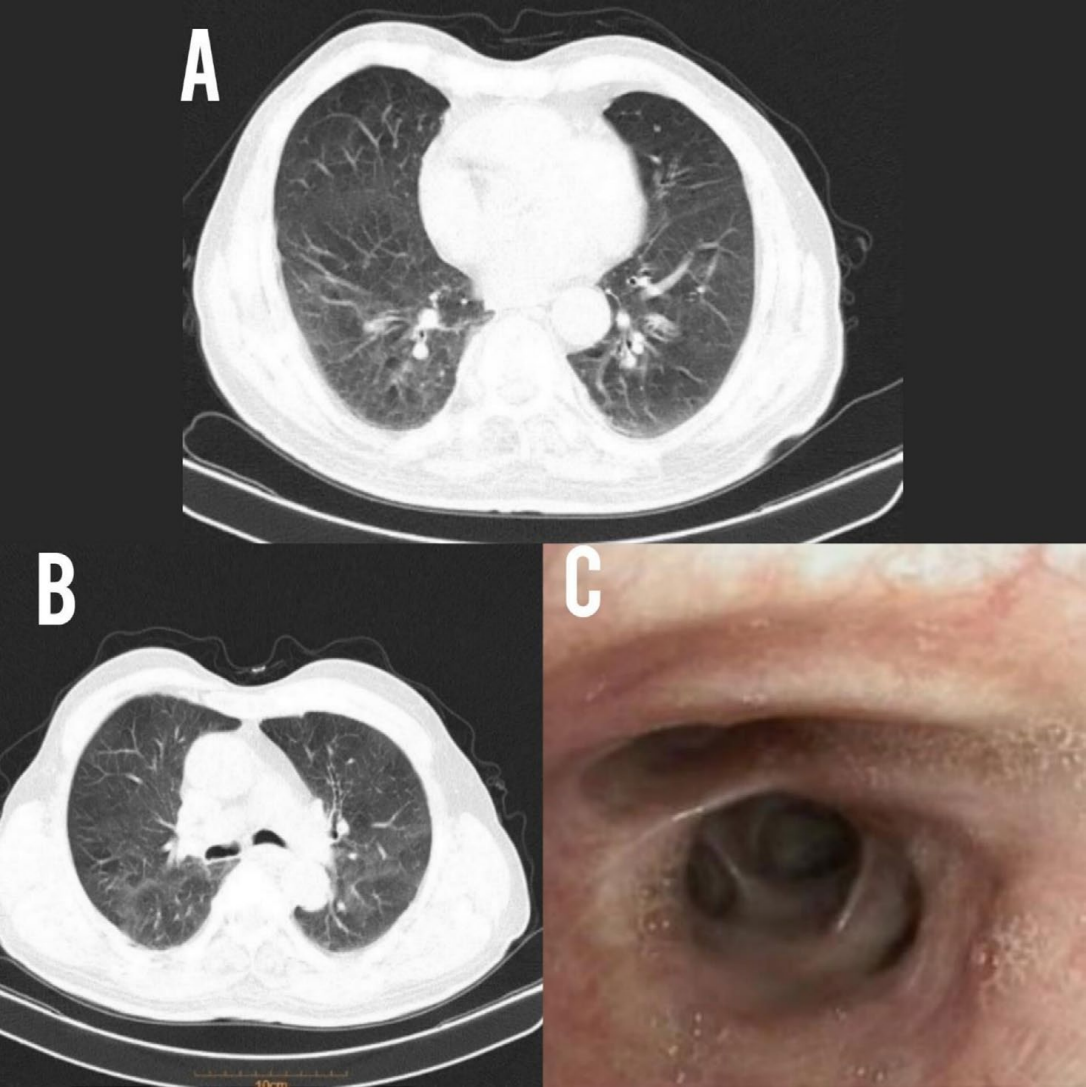
(A and B) Sections of spiral lung CT scan reveal mosaicism and bronchial wall thickening; (C) bronchoscopic view of intermediate bronchus reveals bronchial erythema.

The patient's vital signs and physical examination were unremarkable except for SpO_2_ of 94% and expiratory wheezing. Labs were remarkable for erythrocyte sedimentation rate (ESR) of 38 mm/h (normal reference range: 0–15), C‐reactive protein (CRP) of 17 mg/dL (normal reference range: up to 6) and pH 7.36, PaCO_2_ of 46 mmHg, HCO_3_ 25 mmol/L, PaO_2_ 75 mmHg. Other parts of the lab test such as complete blood count, urine analysis and coagulation studies were within normal range. The patient was admitted and the sputum was sent for microscopy, culture and sensitivity; the results of which showed *Streptococcus* *pneumoniae* . Spiral lung CT scan showed evidence of mosaicism and bronchial wall thickening (Figure 1). Considering non‐life‐threatening hemoptysis, intravenous TXA 500 mg three times a day and nebulized TXA 500 mg three times a day were started for the patient. In addition, the patient was treated with intravenous levofloxacin, corticosteroid and bronchodilators. Fiber‐optic bronchoscopy was performed for the patient, which revealed no evidence of active bleeding; therefore, no TXA was instilled during the procedure.

### 3.2 Case No. 2

**FIGURE 2:**
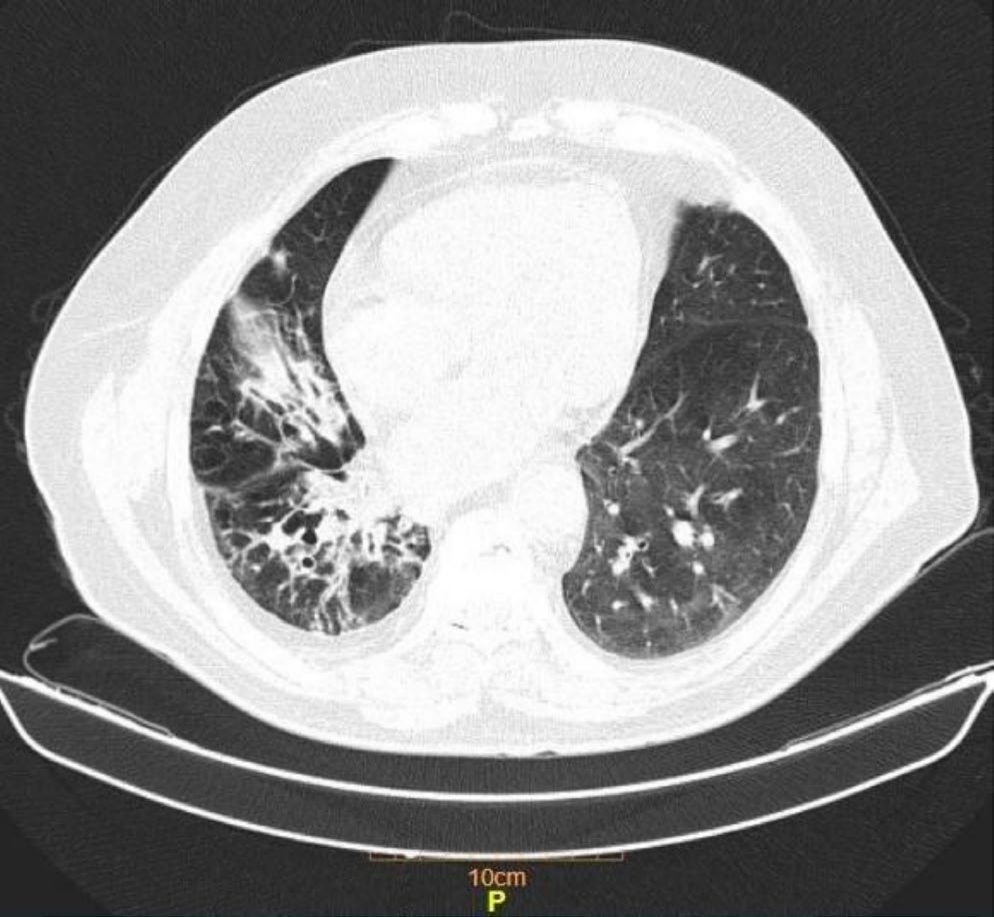
Section of spiral lung CT scan reveals mosaicism, right lower lobe bronchiectasis and trace right‐sided pleural effusion.

Vital signs and physical examination were unremarkable except for expiratory wheezing and right‐sided crackles. Complete Blood Count and biochemical markers were within normal limits except for a white cell count of 11,600/mcL. Inflammatory markers revealed an ESR of 47 mm/h and c‐reactive protein (CRP) of 31 mg/dL (normal reference range: up to 6). The COVID‐19 and influenza PCR results were sent and reported as negative. Sputum gram stain and culture revealed *Pseudomonas aeruginosa* and bronchoalveolar lavage (BAL) AFB stain and GeneXpert were negative. Lung CT scan showed mosaicism and right lower lobe bronchiectasis (Figure 2), and fiber‐optic bronchoscopy revealed no evidence of active bleeding; therefore, no TXA was instilled during the procedure. Antiplatelet drugs were discontinued during the episode of hemoptysis. Meanwhile the patient was managed with TXA 500 mg IV q8h, and 500 mg nebulized TXA q8h, and Tazocin (Piperacillin/Tazobactam) 4/0.5 g IV q6h, corticosteroid and bronchodilators.

## 4 Conclusion and Results (Outcome and Follow‐Up)

### 4.1 Case No. 1

After the first administration of combined nebulized and IV TXA 500 mg every eight hours regimen, bright red blood hemoptysis discontinued. Though clot expectoration continued for 48 h. The IV dose of TXA was halved 24 h after cessation of bright red blood hemoptysis. The IV dose was discontinued after complete discontinuation of hemoptysis, and the nebulized treatment was continued for the remaining days until 5 days keeping a window for antibiotics, steroids, and other supportive measures to take effect. No evidence of any adverse events from TXA was noted from the 5 days course of TXA and the patient was discharged after 7 days. After follow‐up review at 7 and 30 days, no recurrent episode of hemoptysis was demonstrated.

### 4.2 Case No. 2

After the first administration of combined nebulized and IV TXA 500 mg every eight hours, bright red blood hemoptysis discontinued. Though clot expectoration continued for 56 h. The IV dose of TXA was halved 24 h after the cessation of bright red blood hemoptysis. The IV dose was discontinued after the complete discontinuation of hemoptysis, and the nebulized treatment was continued for the remaining days until 5 days keeping a window for antibiotics, steroids, and other supportive measures to take effect. The patient was discharged 8 days after admission with no complications reported and with oral ciprofloxacin for the remaining two days of antibiotic therapeutic duration. After follow‐up review at 7 and 30 days, no recurrent episode of hemoptysis was demonstrated.

## 5 Discussion

Combined route administration of a drug for lung conditions to maximize its therapeutic benefit while reducing adverse effects is not a new concept and has been previously investigated for antibiotic administration, mainly colistin, in the setting of MDR pathogens' lung infections, with promising results [8, 9]. Bearing that in mind, we aimed to describe the use of combined nebulized and systemic TXA in the management of two airway disease patients with hemoptysis and review current reports of using topical TXA in hemoptysis treatment. Nebulized TXA offers a targeted therapeutic approach for non‐life‐threatening hemoptysis, delivering the antifibrinolytic agent directly to the bleeding site in the respiratory tract. This localized action could minimize systemic absorption, reducing the risk of thromboembolic complications (e.g., DVT, stroke) associated with IV or oral TXA particularly in patients who are already at risk of thromboembolic complications [10]. Systemic TXA, while effective, circulates widely, increasing exposure to organs unrelated to the bleeding site. Nebulized TXA could achieve comparable or superior efficacy in controlling bleeding with fewer adverse events, making it ideal for patients with non‐life‐threatening hemoptysis where rapid systemic intervention is unnecessary. However, standardized dosing protocols (typically 500 mg nebulized 1–3 times daily) require further validation through randomized controlled trials.

Life‐threatening hemoptysis on the other hand, demands immediate, aggressive intervention to prevent airway obstruction or hemodynamic collapse. Systemic TXA or endovascular procedures (e.g., bronchial artery embolization) remain first‐line treatments due to their rapid systemic effects or mechanical control. Nebulized TXA alone may be insufficient in this context, as it cannot address proximal bleeding from larger vessels or provide hemostasis quickly enough. However, emerging case reports propose adjunctive use of nebulized TXA alongside systemic therapy, embolization, or endobronchial TXA administration to enhance topical clot stabilization [11, 12]. While theoretically plausible, evidence is sparse, and current guidelines do not endorse nebulized TXA for life‐threatening cases. Further research is needed to evaluate its synergistic role in combination therapies.

Combining both routes may offer theoretical benefits: systemic TXA addresses diffuse bleeding or hyperfibrinolysis, while nebulized TXA targets localized bleeding. In the present study, we reported two cases of non‐life‐threatening hemoptysis that were managed with a combination of nebulized and systemic TXA alongside antibiotics, and corticosteroids, resulting in faster cessation of hemoptysis (after the first dose in both cases). The goal was to achieve a more rapid and complete cessation of bleeding than might be possible with either modality alone, potentially shortening the course of bleeding and avoiding escalation to more potentially invasive procedures.

To the best of our knowledge, no original studies have reported outcomes when both forms are used. The literature on this specific combination is limited to isolated case reports, which similarly reported success. Our cases contribute to this nascent body of evidence suggesting a potential role for combined therapy. However, this approach still raises concerns about cumulative dosing and thrombosis risk, particularly in patients with renal impairment or hypercoagulable states, as the total dosing of TXA has been shown to be directly associated with thrombotic events [13]. Therefore, while the theoretical rationale is compelling, robust RCTs are urgently needed to establish safety profiles, define optimal dosing strategies, and identify which patient populations might benefit most from combined therapy versus monotherapy.

Table 1 demonstrated an overview of similar cases managed globally by nebulized TXA only, or a combination of nebulized and systemic routes. Out of the 30 presented cases, only two cases did not respond to nebulized TXA. Most studies used a 500 mg q6–8h dosing protocol; however, the use of higher and lower doses has also been reported. Also, bronchospasm, pulmonary embolism, and serotonin‐like syndrome were reported as observed side effects. Nevertheless, regarding original studies on nebulized TXA, Schoettler et al. [14] assessed 40 hematopoietic cell transplant recipients diagnosed with diffuse alveolar hemorrhage (DAH) and demonstrated that inhaled TXA resulted in a hazard ratio (HR) of 0.43 (95% CI: 0.19–0.96), and Singleton et al. [15] studied 53 children with hemoptysis on extracorporeal membrane oxygenation (ECMO) and showed a 90.5% success rate in cessation of hemoptysis.

**TABLE 1:** Overview of similar cases of treating hemoptysis with local TXA.

Study	Year	Age	Gender	PMH	Cause of hemoptysis	Severity (massive/non‐massive)	Management	Mortality	Side effect	JBI score
Alghizzawi et al. [16]	2024	90	F	Bronchiectasis, AF	DAPT use	NM	Nebulized TXA	No	No	8
Rushlow et al. [17]	2024	32	M	ESRD	Pneumonia	NR	Nebulized TXA + Cryotherapy	No	No	7
Kazi et al. [18]	2024	57	F	HTN, T2DM	GPA	M	Nebulized TXA + ECMO + Solumedrol IV + Corticosteroid	No	No	7
Epler et al. [12]	2024	44	M	Rib aspergillus osteomyelitis, CKD, Pan‐uveitis	NR	NM	Endobronchial TXA	No	SLS	8
Vallabhajosyula et al. [19]	2024	69	M	Gout	Angiosarcoma	NM	Inhaled TXA	No	No	8
Lachute et al. [20]	2024	45	M	TB	Rasmussen's aneurysm	M	Nebulized TXA + artery embolization	Yes	No	8
Fiore et al. [21]	2024	74	F	Chronic respiratory failure requiring tracheostomy	Atypical pneumonia	NM	Nebulized TXA	No	PTE	8
Clarke et al. [22]	2023	47	M	COPD, T2DM, AKI	NR	M	Inhaled TXA (unresponsive) + left bronchial artery embolization + endobronchial valve	No	No	8
Yazdi et al. [23]	2023	78	M	SCC of piriform sinus	Metastasis	M	Nebulized TXA for 7 days + APC	No	No	7
Babalola et al. [24]	2023	25	M	BPD, Epilepsy	EVALI	NM	Nebulized TXA	No	No	8
Roy et al. [25]	2023	52	M	AC	Tracheal hair growth irritation	NM	Nebulized TXA	No	No	6
Joshi et al. [26]	2023	55	F	Cirrhosis	Cirrhosis‐induced coagulopathy	NM	Inhaled TXA + Cryoprecipitate	No	No	8
Pusukur et al. [27]	2023	61	F	COPD, Esophageal varices, pulmonary *Mycobacterium szulgai*	Rasmussen's aneurysm	M	Inhaled TXA (unresponsive) + embolization of the pseudoaneurysm	No	No	7
Grant‐Sittol et al. [28]	2022	82	F	CAD, HTN	Vasculitis	M	Inhaled TXA 500 mg q8h for 3 days	Yes	No	8
Wu et al. [29]	2022	34	M	SLE	SLE	M	Inhaled TXA + corticosteroid + plasmapheresis	No	No	8
Agustin et al. [11]	2020	38	M	NR	Left bronchial varix	M	Nebulized TXA + IV TXA	No	No	7
Alabdrabalnabi et al. [2]	2020	66	F	CAD, Rheumatic heart disease	NR	NR	100 mg Nebulized TXA q8h (responded after 5 days) + 500 mg IV TXA + 1 unit PC + 6 unit FFP	No	No	8
Azharuddin et al. [30]	2019	70	M	COPD, *Mycobacterium xenopi*	*Mycobacterium xenopi* cavitation	NM	500 mg Nebulized TXA q8h	No	No	8
Sanghvi et al. [31]	2019	78	F	HTN, DLP, AF, Stroke	tPA administration	NR	2000 mg Nebulized TXA	No	No	8
Komura et al. [32]	2018	69	F	Lung AC stage IV	Lung AC	M	1000 mg Nebulized TXA (responsive after 10 min)	No	No	8
Abdelkader et al. [33]	2018	70	M	HCC with lung metastasis	Metastasis	NR	500 mg Nebulized TXA (responded immediately)	No	No	5
		77	F	COPD, HF, CKD	Post bronchoscopy	NR	500 mg Nebulized TXA	No	No	
Calvo et al. [34]	2016	58–84	4 M	Lung cancer, bronchiectasis	Lung cancer, bronchiectasis	NM	250–500 mg Nebulized TXA q8‐12h (responded after 6‐48 h)	No	Bronchospasm	5
Hankerson et al. [35]	2015	46	M	Piriform sinus cancer + Thyroid SCC	Invasive laryngotracheal tumor	NM	1000 mg Nebulized TXA	No	No	8
Solomonov et al. [5]	2009	67	M	RCC, lung metastasis	Bronchoscopic Bx	M	500 mg endobronchial TXA (immediate response)	No	No	8
		43	M	MTC, lung metastasis, PHTN	Metastasis	NR	500 mg aerosolized TXA q6h (response after 20 min)	No	No	
		49	F	MF, ITP, AML	DAH	NR	500 mg aerosolized TXA q6h (response after 3 h)	No	No	
		52	M	Lymphoma	Metastasis	M	500 mg aerosolized TXA q6h	Yes	No	
		57	M	NR	Bronchoscopic Bx	M	1000 mg endobronchial TXA	No	No	
		59	M	Vitrectomy	Idiopathic	NR	500 mg aerosolized TXA q6h (immediate response)	No	No	

Abbreviations: AC, adenocarcinoma; AF, atrial fibrillation; AKI, acute kidney injury; AML, acute myeloid leukemia; APC, argon plasma coagulation; BPD, bipolar disease; Bx, biopsy; CAD, coronary artery disease; CKD, chronic kidney disease; COPD, chronic obstructive pulmonary disease; DAH, diffuse alveolar hemorrhage; DAPT, dual antiplatelet therapy; DLP, dyslipidemia; ECMO, extracorporeal membrane oxygenation; ESRD, end‐stage renal disease; EVALI, E‐cigarette and vaping associated lung injury; GPA, granulomatosis with polyangiitis; HCC, hepatocellular carcinoma; HF, heart failure; HTN, hypertension; ITP, immune thrombocytopenic purpura; IV, intravenous; JBI, Joanna Briggs Institute Critical Appraisal Tool; MF, myelofibrosis; MTC, medullary thyroid carcinoma; NR, not reported; PTE, pulmonary thromboembolism; RCC, renal cell carcinoma; SCC, squamous cell carcinoma; SLE, systemic lupus erythematous; SLS, serotonin‐like‐syndrome; T2DM, type 2 diabetes mellitus; TB, tuberculosis; TXA, tranexamic acid.

This case report describes a novel therapeutic approach for the control of hemoptysis through the concurrent use of intravenous and nebulized tranexamic acid, a combination that, to our knowledge, has not been previously reported for patients with airway diseases. Its clinical significance is multifactorial. First, it presents a potential alternative for high‐thrombotic‐risk patients in whom antiplatelet or anticoagulant therapy may also need to be discontinued because of the extent of hemoptysis. Second, for patients with airway diseases lacking a discrete endobronchial lesion amenable to bronchoscopic intervention, this combined route may act as a systemic and topical hemostatic bridge, allowing time for medical and other supportive measures (e.g., antibiotics, steroids) to take effect. Although well‐designed clinical trials can evaluate the therapeutic effects in patients with negative microbiological studies. Finally, in life‐threatening hemoptysis, it could serve as a vital stabilization and bridging strategy prior to definitive interventional procedures (such as bronchoscopic intervention or angio‐embolization). These potential applications highlight the need for further rigorous investigation.

One significant limitation of this study is the challenge in conclusively attributing clinical improvements solely to nebulized TXA. As all participants received standard care, including antibiotics, corticosteroids, supportive therapy, and other interventions tailored to their underlying conditions, the observed benefits may reflect the cumulative effect of conventional treatments rather than the TXA specifically. The absence of a control group receiving *only* standard therapy precludes definitive causal inference. This design limitation underscores the possibility that the natural course of recovery or primary treatments drove the outcomes. To address this gap, rigorously designed randomized controlled trials or longitudinal observational studies are essential. However, a more promising effect was the discontinuation of bright red blood hemoptysis after the first dose administration of combined nebulized and IV TX, rather than the complete discontinuation of hemoptysis after a few days, a favorable issue that needs to be further investigated in future studies. Further studies are also suggested to directly compare nebulized TXA as an add‐on therapy to standard care alone, using a well‐matched control group.

## 6 Conclusion

Nebulized TXA may demonstrate efficacy as a localized, minimally invasive therapy for non‐life‐threatening hemoptysis, offering rapid bleeding cessation and reduced systemic side effects compared to traditional systemic routes. Also, combined nebulized and systemic TXA may synergistically address bleeding, though cumulative dosing risks require further investigation. Standardized protocols and robust clinical trials are essential to validate safety, optimize dosing, and define its role in life‐threatening hemoptysis or high‐risk populations.

## Author Contributions

**Erfan Ghadirzadeh:** conceptualization, methodology, project administration, visualization, writing – original draft, writing – review and editing. **Faezeh Shokri:** data curation, writing – original draft, writing – review and editing. **Shamim Khorshidian:** data curation, writing – original draft, writing – review and editing. **Anita Ziari:** data curation, visualization, writing – original draft, writing – review and editing. **Mobina Gheibi:** data curation, software, writing – review and editing. **Majidreza Adelani:** data curation, visualization, writing – original draft, writing – review and editing. **Seyed Pooria Salehi Mashhad Sari:** data curation, writing – original draft, writing – review and editing. **Fatemeh Varshoei:** conceptualization, methodology, writing – review and editing. **Hossein Mehravaran:** conceptualization, methodology, resources, supervision, validation, writing – review and editing.

## Ethics Statement

This study was approved by the ethical committee of Mazandaran University of Medical Sciences (No. IR.MAZUMS.REC.1404.076).

## Consent

The authors declare that written informed consent was obtained for the publication of this manuscript and accompanying images using the form provided by the Journal.

## Conflicts of Interest

The authors declare no conflicts of interest.

## Data Availability

The data are available with the corresponding author and can be reached on request.
